# Investigation of Chemical Composition, Antioxidant Activity, and the Effects of Alfalfa Flavonoids on Growth Performance

**DOI:** 10.1155/2020/8569237

**Published:** 2020-02-10

**Authors:** Si Chen, Xiang Li, Xin Liu, Ning Wang, Qi An, Xi Mei Ye, Zi Tong Zhao, Meng Zhao, Yi Han, Ke Hui Ouyang, Wen Jun Wang

**Affiliations:** ^1^Jiangxi Province Key Laboratory of Animal Nutrition/Engineering Research Center of Feed Development, Jiangxi Agricultural University, Nanchang 330045, China; ^2^Jiangxi Key Laboratory of Natural Product and Functional Food, College of Food Science and Engineering, Jiangxi Agricultural University, Nanchang 330045, China

## Abstract

The flavonoids were extracted from alfalfa using ethanol assisted with ultrasonic extraction and purified by D101 macroporous resin column chromatography. The chemical composition and content of ethanol elution fractions (EEFs) were assessed by ultrahigh-performance liquid chromatography and hybrid quadrupole time of flight mass spectrometry (UHPLC-Q-TOF-MS) and aluminum nitrate-sodium nitrite-sodium hydroxide colorimetric method. The *in vitro* antioxidant activity of two EEFs was conducted by scavenging DPPH free radical, and the main antioxidants of 75% EEFs were screened using DPPH-UHPLC. Moreover, the *in vivo* antioxidant activity of 75% EEFs and the growth performance of broilers were studied. The results showed that the content of 30% and 75% EEFs was 26.20% and 62.57%. Fifteen compounds were identified from 75% EEFs, and five of them were reported in alfalfa for the first time. The scavenging activity of 75% and 30% EEFs (200 *μ*g/mL) against DPPH was 95.51% and 78.85%. The peak area of 5,3′,4′-trihydroxyflavone and hyperoside was decreased by 82.69% and 76.04%, which exhibited strong scavenging capacities. The total antioxidant capacity (T-AOC), superoxide dismutase (SOD), and glutathione peroxidase (GSH-PX) level of three treated groups against the normal control group (NC) fed with basal diet significantly increased by 3.89-24.49%, 0.53-7.39%, and 0.79-11.79%, respectively. While the malondialdehyde (MDA) decreased by 0.47-18.27%. Compared with the NC, the feed to gain ratio (F : G) of three treated groups was lowered by 2.98-16.53% and survival rate of broilers significantly increased. Consequently, 75% EEFs extracted from alfalfa exhibited powerful antioxidant activities and might be a potential feed additive to poultry and livestock.

## 1. Introduction

Oxidation is an essential biological process central to the functioning of living organism for energy production. However, it also leads to the production of reactive oxygen species (ROS), which may result in lipid oxidation, protein damage, and even cell death [1]. Antioxidant plays an important role in delaying, preventing, and removing peroxide products and has been used to lessen the impact on oxidative damage [2]. Synthetic antioxidants, with the characteristics of low cost, high performance, and broad bioactivity, have long been in common usage in food, pharmaceutical and cosmetics, health products, and animal feed industry. Nevertheless, it has been reported that synthetic antioxidants are restricted due to their potential carcinogenicity [3]. Hence, there has been more and more interests in developing and using natural, effective, safe, and multiple biological antioxidants to replace synthetic antioxidants in many industries, such as natural plant polyphenol compounds [4, 5]. The flavonoids are a group of polyphenol compounds with a basic structure of C6-C3-C6 carbon skeleton and known for powerful antioxidant capacities based on their polyhydroxyl structures [6, 7].

Alfalfa, with the reputation of “king of herbage,” is a high-yield perennial leguminous plant that is mostly grown as food for farm animals and has been cultivated in China for more than two thousand years [8]. In recent years, some studies have reported that alfalfa contained flavonoids, saponins, dietary fiber, vitamins, minerals, organic acids and polysaccharide [9–11]. The above-mentioned bioactive substances have possessed a wide range of bioactivities such as antioxidant, anti-inflammation, anticancer, and enhancing immunity function [12–16]. Hence, alfalfa flavonoids have been usually used as an additive added in animal feed to promote the antioxidant activity of serum and liver, meat quality, growth, and production performance. Jing et al. [17] reported that the flavonoids derived from alfalfa had exhibited very strong antioxidant activity. The *in vitro* antioxidant capacities determined by DPPH and ABTS assay were 80.72% and 87.38%. Zhan et al. [18] had investigated the effects of alfalfa flavonoids on production performance, immunity, and ruminal fermentation of dairy cows. The results revealed that the flavonoids could sharply reduce methane dicarboxylic aldehyde concentration, and the superoxide dismutase enzyme activity presented a significant increasing tendency. Xie et al. [19] reported that the exposure of alfalfa to hydrogen-rich water (HRW) could increase the flavonoid content. *In vitro* tests showed that the flavonoids activated the total and isozymatic activities of SOD, POD, and CAT. Ouyang et al. [20] had found that alfalfa flavonoids enhanced the *in vivo* antioxidant activities (such as SOD, CAT, T-AOC, and GSH-PX) and decreased the level of MDA in serum. In addition, the average daily gain and body weight of the broilers fed with flavonoids were higher than those of broilers fed with basal diet. Chen et al. [21] reported that the average daily gain of Yangzhou geese fed with alfalfa flavonoids was significantly higher than that of the control group fed with basal diet and the blood biochemical indices could be increased. However, there have been currently few studies about the identification of alfalfa flavonoids and analysis of dominant *in vitro* antioxidant by DPPH^·^-UHPLC.

The objective of this report was to investigate the effect of alfalfa flavonoids on *in vitro* (DPPH radical scavenging) and *in vivo* antioxidant activity (SOD, T-AOC, GSH-PX, and MDA) of broiler serum. The chemical compositions of 75% EEFs from alfalfa were identified with UHPLC-Q-TOF-MS/MS, and the dominant *in vitro* antioxidant was investigated. Meanwhile, the growth performance and mortality of broilers were analyzed in order to explore the feasibility of alfalfa flavonoids as a natural plant antioxidant in poultry and livestock industries.

## 2. Materials and Methods

### 2.1. Materials

The aerial parts of alfalfa was provided by grassland base of Jiangxi Grassland Station (Nanchang, Jiangxi) and then dried in an oven for 24-48 hours at 65°C, smashed with a mini-type disintegrator. The alfalfa powder was filtered through a 60 mesh sieve and kept at -40°C for following research. Standards of rutin, apigenin, isoquercitrin, luteoloside, quercetin, and kaempferol were purchased from Beijing Solarbio Science & Technology Co., Ltd (Beijing, China). 1,1-Diphenyl-2-picrylhydrazyl (DPPH), sodium nitrite (NaNO_2_), aluminum nitrate (Al(NO_3_)_3_, 9H_2_O), and sodium hydroxide (NaOH) were purchased from Aladdin Biotechnology Co., Ltd. (Shanghai, China). Absolute ethanol, ethyl acetate, acetonitrile, and methanol were purchased from TEDIA Company Inc. (Shanghai, China), and ultrapure water (18.2 M*Ω* resistivity) was obtained from the Milli-Q system (Millipore, Bedford, MA, USA). The MDS, SOD, T-AOC, and GSH-PX assay kits were purchased from Nanjing Jiancheng Bioengineering Institute (Nanjing, Jiangsu, China). All organic reagents were either HPLC or analytical grade.

### 2.2. Extraction and Purification of Alfalfa Flavonoids

Alfalfa flavonoids were extracted and purified according to Xu et al. [22] with slight modifications. In brief, the procedures were as follows: the alfalfa powder was soaked in 20% aqueous ethanol (*v*/*v*) overnight. After that, the anhydrous ethanol was added into the soak solution to obtain 75% ethanol solution, which was pretreated at 50°C with robust stirring for an hour. The presoak solution was put into ultrasonic extracting tank with the ultrasonic time of four hours and ultrasonic power of 70 W. The extracting solution was filtered, and the filter residue was extracted twice more by following the same steps as above. Then, three extracting solutions were filtered with gauze, and all the filtrates were mixed. Several hours later, the lower layer was separated by a separating funnel and evaporated under a vacuum at 40°C to yield the crude extract, which was further dissolved in water and partitioned with ethyl acetate. The ethyl acetate phase was evaporated to near dryness, which was further suspended in water and then separated by D101 macroporous resin column. The gradient elution mode was ethanol/H_2_O (0 : 100, 30 : 70, 75 : 25, *v*/*v*) at a flow rate of 1 mL/min. When the color of the eluent was colorless, it indicated that the gradient elution had completed. All of the fractions eluted were, respectively, concentrated by rotary evaporator under a vacuum at 50°C, and then, the concentrated extracts were stored in a freezer at -80°C for twelve hours. At last, the extracts were made into dry powder by vacuum freeze-drying technology. However, the water extract was too few to be collected and abandoned. The 30% and 75% EEFs were collected with self-sealing plastic bags and stored in a cool and dry place for further experiment.

### 2.3. Determination of Total Flavonoid Content (TFC) of EEFs

The TFC of 30% and 75% EEFs were determined on the basis of the method by Chen et al. [23]. The procedure was as follows: the rutin standard was accurately weighed into a 100 mL volumetric flask and dissolved in 75% aqueous ethanol in order to obtain the standard stock solution with the concentration of 0.40 mg/kg. Then, the standard stock solution was gradually diluted with 75% aqueous ethanol to obtain five running solutions containing 0 (blank sample), 0.016, 0.032, 0.048, 0.056, and 0.08 mg per 1 mL, respectively. Each of the EEFs and rutin running solutions was conducted by the following steps: 0.5 mL of EEFs or rutin standard solution and 0.15 mL of 5% sodium nitrite were mixed. Six minutes later, 0.15 mL of 10% aluminum nitrate was added. In the next six minutes, 2 mL of 4% sodium hydroxide solution was poured in the above mixture. After shaking well and three minutes, optical density of the reaction solution was measured by a microplate reader at 510 nm and recorded against the blank sample. The TFC of two EEFs were calculated using the standard curve of rutin established by the relationship between optical density and concentration. Each sample was measured three times.

### 2.4. *In Vitro* Antioxidant Activity

In our work, the in vitro antioxidant activity was evaluated by the DPPH free-radical assays. The DPPH radical-scavenging activity was performed as described by Schaich et al. [24]. Specifically, each of the different concentrations (25-200 *μ*g/mL) of 30% EEFs, 75% EEFs, rutin standard was mixed (1 : 1, *V*/*V*) respectively with DPPH solution (0.1 mmol/L), and the optical density was measured at 517 nm after 25 min of incubation in the dark at room temperature. The free-radical scavenging of DPPH was calculated according to the following equation:

(1) $$\begin{array}{c} \text{Scavenge rate }\left(\%\right)=\left[\frac{A_{0}–A_{i}+A_{j}}{A_{0}}\right]\times =100, \end{array}$$

where *A*_0_ was the optical density of mixing ultrapure water and DPPH after 25 min; *A*_*i*_ was the optical density of mixing samples/rutin and DPPH after 25 min; *A*_*j*_ was the optical density of mixing sample/rutin with anhydrous alcohol after 25 min. The DPPH free-radical scavenge rate of two EEFs was compared to the positive control (rutin). All experiments were conducted in triplicates, and the results were presented as the mean ± standard deviation.

### 2.5. UHPLC-Q-TOF-MS/MS Conditions

The UHPLC-MS was carried out on a 5600 accurate mass tandem quadrupole time-of-flight mass spectrometer (AB SCIX, U.S.A.), which was equipped with a LC30 system (Shimadzu, Japan) including a pump, autosampler, and column oven. The chromatographic separation was conducted on Shim-pack GIST C18 column (2.1∗75 mm, 2 *μ*m, Shimadzu, Japan), the mobile phase consisted of solvent A (0.1% formic acid aqueous solution) and acetonitrile as solvent B. The gradient elution program was as follows: 0-3 min, A : B (95 : 5, *V*/*V*); 3-7 min, A : B (90 : 10, *V*/*V*); 7-45 min, A : B (80 : 20, *V*/*V*); 45-70 min, A : B (68 : 32, *V*/*V*); 70-76 min, A : B (30 : 70, *V*/*V*); and 76-83 min, A : B (95 : 5, *V*/*V*). The total run time was 83 min at a flow of 0.2 mL/min with the column temperature maintained at 40°C. The injected volume of samples was 10 *μ*L, and the column effluent was subjected to a electrospray ionization source (ESI) that was performed in negative ion mode, nebulizer pressure, curtain gas, 40 psi; nebulizer gas, 50 psi; heater gas, 50 psi; collision energy, -10 eV (MS), −30 ± 15 eV (MS/MS) eV; declustering potential, -80 V; ion spray voltage floating, -4.5 kV; and turbo spray temperature, 550°C. The mass spectrometry spectra were collected at the range of *m*/*z* 50-1000.

### 2.6. DPPH-UHPLC Experiment

The DPPH free-radical-scavenging activity was performed as described above. In brief, 75% EEFs (1.0 mg/mL) and DPPH (0.1 mmol/L) were mixed 1 : 1 (*V*/*V*) at room temperature for 25 min, and then, the reaction mixtures were passed through a 0.45 *μ*m organic membrane filter immediately before UHPLC analysis. The 75% EEFs (0.5 mg/mL) without reacting with DPPH was used as the control. The DPPH-UHPLC conditions were the same as mentioned above. The reduction of peak areas of a substance was indicated that the content of this substance was reduced. In other words, the more the reduction of peak area was, the stronger the ability to scavenge free would be.

### 2.7. Ultrahigh Performance Liquid Chromatography (UHPLC) Conditions

The separation column was Shim-pack GIST C18 (2.1^∗^75 mm, 2 *μ*m). The UHPLC system was equipped with a diode array detector (DAD), an autosampler, a pump, and a column compartment. The injection volume was 10 *μ*L, and the detection wavelength was 340 nm. Other chromatographic conditions were the same as those of UHPLC-MS mentioned above, including solvent A, solvent B, gradient elution program, flow rate, and column temperature. The calculation of peak area was carried out by Shimadzu chromatography workstation.

### 2.8. Experimental Design of Animals

Two hundred and fifty 1-day-old healthy broilers (Chongren Spotted Chickens, Jiangxi, China) vaccinated against infectious bronchitis and Newcastle disease were purchased from a local chicken farm. At the beginning of preexperiment, all the three-tier ladder cages and chicken house were cleaned and strictly disinfected. Two hundred healthy broilers (half male and half female) with similar body weight, aged about 2 weeks at the start of the experiment, were used and divided randomly into the NC and low-dose group (LDG), middle-dose group (MDG), and high-dose group (HDG) with 5 replicates per group and 10 broilers per replicate. The NC was fed with the basal diet, and LDG, MDG, and HDG were fed with the basal diet supplemented with 200, 300, and 400 mg/kg 75% EEFs, respectively. The feeding trial lasted 56 d, and the daily diet was divided into two stages: a starter diet from 0 to 28 d and a grower diet from 29 to 56 d. The starter diet included 21.24% crude protein, 12.67 MJ/kg metabolic energy, 3.12% fiber, 7.06% crude fat, 0.98% Ca, 0.43% P, 0.20% NaCl, 1.28% Lys, 0.57% Met, and 0.81% Met+Cys, and the grower diet included 20.01% crude protein, 12.82 MJ/kg metabolic energy, 3.01% fiber, 8.42% crude fat, 0.92% Ca, 0.42% P, 0.16% NaCl, 1.18% Lys, 0.46% Met, and 0.76% Met+Cys. The boilers were raised in the three-tier ladder cages equipped with fresh water tube and feed channel and allowed free access to the water and feed. The experiment was implemented following the normal vaccination procedure, and the room temperature of the chicken house was maintained at about 35°C for 0-3 days, 32°C for 4-5 days, and 30°C for 6-7 days and then reduced by 2°C each week until the temperature was reached and was maintained at 24°C at the rest of the animal feeding trial. The photoperiod of broilers was set for 24 h light for the first 4 d, 20 h light : 4 h dark for 5-9 d and 16 h light : 8 h dark thereafter. Approval of all the procedures and related protocols adopted in this experiment was obtained from the Institution Animal Ethics and the Animal Care and Use Committee of Jiangxi Agricultural University (Jiangxi, China).

#### 2.8.1. Growth Performance and Mortality Rate Measurement

Each broiler in all the cages was weighed at the age of 1 d, 28 d, and 56 d in order to calculate the average daily gain (ADG). Feed consumption on a cage basis was weighted and recorded daily to be calculated. The feed to gain ratio (F/G) was calculated according to the equation: F/G = the average daily feed intake (g)/the average daily gain (g). The death rate of broilers on a cage basis was calculated by the formula: death rate (%) = (number of dead broiler/number of experimental broiler)∗100.

#### 2.8.2. The Isolation of Broiler Serum

Four broilers of each cage/replicate (half male and half female) were selected randomly after fasting for the age of 28 d and 56 d, and then, the blood samples (about 4 mL) were collected from wing vein into heparin anticoagulant tubes. The tubes were kept for one hour at the room temperature and then centrifuged for 20 min at 3500 rpm. The upper broiler serum was transferred into five clean tubes (100 *μ*L per tube) and stored in the -80°C refrigerator for the following analysis.

#### 2.8.3. The Analysis of *In Vivo* Antioxidant Activities

The levels of SOD, T-AOC, and GSH-PX in broiler serum were measured by the commercial WST-1, ABTS, and colorimetric assay kit; the concentration of MDA was detected by the TBA assay kit (Nanjing Jiancheng, China). All of these operations were implemented in accordance with the statement of kit instructions. All the experiments were performed in triplicates.

### 2.9. Statistics and Data Analysis

All data was performed by IBM SPSS statistics 20 software and analyzed by a least significant difference (LSD) with a confidence interval of 95% used to compare the mean, and the results were presented as the mean ± standard deviation (SD). The graphs were drawn with Origin 9.0.

## 3. Results

### 3.1. Determination of Total Flavonoid Content (TFC) of EEFs

**Figure 1:**
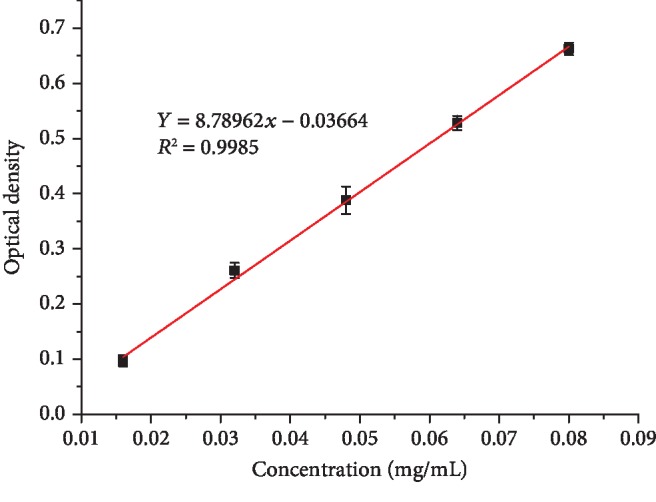
The standard curve of rutin.

In our work, the NaNO_2_–Al(NO_3_)_3_ colorimetric methods were used to detected TFC and the optical density (OD) of 75% and 30% EEFs were 0.51 and 0.19. The standard curve of rutin is shown in Figure 1. Then, the content of 30% and 75% EEFs was calculated by the formula: *Y* = 8.78962*x* − 0.03664 and the results were 26.20% and 62.57%, respectively.

### 3.2. Determination of *In Vitro* Antioxidant Activity

**Figure 2:**
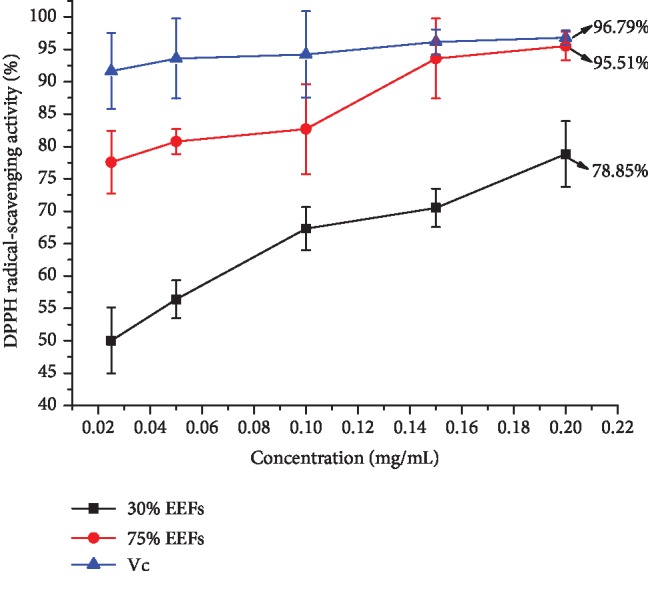
The DPPH free-radical-scavenging rate of Vc, 30% EEFs, and 75% EEFs.

The aim of our work was to select a representative DPPH free radical to determine the antioxidant capacity of 30% and 75% EEFs, and vitamin c (Vc) was used as the positive control. The result is presented in Figure 2. It can be seen that the DPPH^·^ scavenging rate increased with the increase of the content of alfalfa flavonoids. The 30% and 75% EEFs exhibited certain antioxidant activities. The scavenging activities of 75% EEFs were higher than those of 30%. In particular, the scavenging rate of 75% and 30% EEFs (200 *μ*g/mL) was 95.51% and 78.85% and the Vc (positive control) was 96.79%.

### 3.3. Identification of Flavonoids

**Figure 3:**
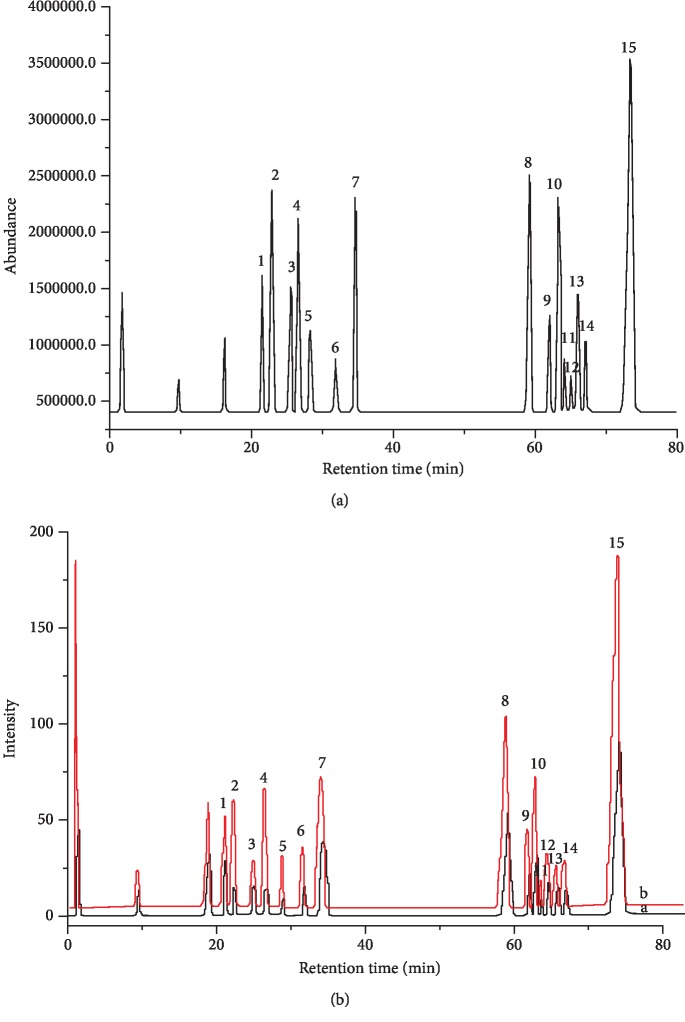
The DPPH-UHPLC (a) and UHPLC chromatogram (340 nm) (b) of 75% EEFs.

The mass spectrometry of 75% EEFs is exhibited in Figure 3(a), and peak assignments are shown in Table 1. As shown in Table 1, the structures of 1, rutin; 2, hyperoside; 3, astragali; 4, 5,3′,4′-trihydroxyflavone; 5, chrysoeriol-7-0-glucoside; 10, tricin; 6, liquiritigenin; 7, 7,4′-dihydroxyflavone; 13, formononetin; 9, chrysoeriol; 14, isoliquiritigenin; 15, 7,4′-dimethoxy-5-hydroxyflavanone; 12, 3′,7-dimethoxy-3-hydroxyflavone; 11, limocitrin; and 8, apigenin were confirmed by the related literature, standard of HPLC retention time and MassBank, five of which were reported for the first time in alfalfa, including hyperoside, liquiritigenin, limocitrin, 7,4′-dimethoxy-5-hydroxyflavanone, and isoliquiritigenin. The MS/MS^2^ data of compound 1 included parent ions [M-H]^−^ at the *m*/*z* value of 609.1487 (C_27_H_30_O_16_) with the fragmentation at the *m*/*z* value of 300.0242 [M-H-146-162]^−^ (successive loss of rhamnosyl and glucosyl) [23].

Compound 2 presented parent ion [M-H]^−^ at the *m*/*z* value of 463.0883 (C_21_H_19_O_12_) with the fragmentation at the *m*/*z* value of 301.0343/300.0261 [M-H-162]^−^ which was resulted from the hexoside cleavage, and then, the [M-H]^−^ (*m*/*z* 300.0261) ions further generated [M-H-162-28]^−^ at the *m*/*z* value of 271.0282 and [M-H-162-44]^−^ at the *m*/*z* value of 255.0289 for the loss of CO and CO_2_ in the MS^2^, respectively [25].

Compound 3 emerged precursor ions [M-H]^−^ (*m*/*z* 447.1630) according to the pyrolysis of flavonoid glycosides, and then, the parent ions produced the fragmentation [M-H-C_6_H_11_O_5_]^−^ (*m*/*z* 284.0343) due to the lost of a hexoside. The product ions at *m*/*z* 255.0350 and *m*/*z* 227.0431 were obtained from fragmentation of the [M-H-C_6_H_11_O_5_]^−^ (*m*/*z* 284.0343) corresponding to losses of the CHO and CHO, CO in negative ion mode [26].

It could be observed that compound 4 shared the molecular ions at [M-H]^−^*m*/*z* 269 with 8. However, the predominant ions of compound 4 appeared at *m*/*z* 133.0295 (C_8_H_5_O_2_) and *m*/*z* 135.0087 (C_7_H_3_O_3_) based on the homolytic cleavage that resulted in the opening of C ring. According to the Retro-Diels-Alder (RDA) theory, the hexacyclic compounds with double bonds might be decomposed into fragmentary ions of diene and dienophile under the effect of ion source. Therefore, compound 8 could fragment by (RDA) reaction, which generated fragment ions at *m*/*z* 117.0345 (C_8_H_5_O) and *m*/*z* 151.0036 (C_7_H_3_O_4_) owning to the opening of C ring. Analysis of related literature [27] and the MassBank, compounds 4 and 8 were identified as 5,3′,4′-trihydroxyflavone and apigenin, respectively.

The molecular ion of compound 5 appeared at [M-H]^−^ at *m*/*z* 461.1090 (C_21_H_19_O_12_) and characteristic fragment ion at [M-H-162]^−^*m*/*z* 283.0282 which further fragmented into product ions at [M-H-162-28]^−^ (*m*/*z* 255.0341) due to losses of aglycone fragments and CO [28].

Compounds 6 and 14 had the same precursor ions [M-H]^−^ at *m*/*z* 255 and fragment ions at [M-H-120]^−^*m*/*z* 135.01 and [M-H-120-44]^−^*m*/*z* 91.02 while the retention times were 32.81 and 67.04 min, which indicated that they were isomers. According to the polarity and elution order of two compounds, compounds 6 and 14 were identified as liquiritigenin and isoliquiritigenin, respectively, which agree with the literature [29].

Compound 7, with the typical mother nucleus of flavone, was very prone to the RDA reaction, which could yield molecular ions [M-H]^−^ at *m*/*z* 253.05 and further produced fragment ions at [M-H-118]^−^*m*/*z* 135.01 (C_7_H_3_O_3_), *m*/*z* 117. 04 (C_8_H_5_O), and [M-H-118-44]^−^*m*/*z* 91.02 coupled with the C ring opening and a loss of CO_2_.

Compounds 9 and 15 presented the same ions [M-H]^−^ at *m*/*z* 299, [M-H-CH_3_]^−^ at *m*/*z* 284, and [M-H-CH_3_-CO]^−^ at *m*/*z* 256 but different fragment ions [M-H-28]^−^ at *m*/*z* 271.0623. Taking into account their structures, MS/MS^2^ data, and the study reported previously [30], it was deduced that the C ring of compound 9 had a C_2_, C_3_ double bond, which formed a conjugated near-plane structure with A and B ring, whereas the compound 15 lack of a stable conjugated system had a trend to lose a CO. Consequently, structure of 9 was tentatively identified as chrysoeriol [31] while 15 could be assigned as 7,4′-dimethoxy-5-hydroxyflavanone.

Compound 10 displayed molecular ions [M-H]^−^ at *m*/*z* 329.0691, which gave fragment ions [M-H-CH_3_]^−^ at *m*/*z* 314.0453, [M-H-CH_3_-CH_3_]^−^ at *m*/*z* 299.0211, and [M-H-CH_3_-CH_3_-CO]^−^ at *m*/*z* 271.0273 in MS^2^. Compared with the precious publications [32], structure of 10 was regarded as tricin.

Compound 11 exhibited molecular ions [M-H]^−^ at *m*/*z* 345.2276. Based on the RDA, the precursor ions further generated the fragment ions [M-H-15]^−^ at *m*/*z* 330.0469 and [M-H-30]^−^ at *m*/*z* 315.0121 [33].

The fragment ions of compound 12 were produced mostly by the loss of a CH_3_ and CO_2_. The fragment ions included [M-H]^−^ at *m*/*z* 297.0412, [M-H-17]^−^ at *m*/*z* 282.0199, and [M-H-44]^−^ at *m*/*z* 253.0149.

Compound 13 could generate the ions [M-H]^−^ at *m*/*z* 267.0665, [M-H-CH_3_]^−^ at *m*/*z* 252.0434, [M-H-O]^−^ at *m*/*z* 251.0349, [M-H-CO_2_]^−^ at *m*/*z* 223.0393, and [M-H-CO_2_-CO]^−^ at *m*/*z* 195.0454 at low and high collision energy. The intensity of ions at *m*/*z* 252.0434 was stronger than that of ions at *m*/*z* 251.0349, which was obviously different from the distribution of isotope ions. Hence, the ions at *m*/*z* 252.0434 and 251.0349 were all assigned as the fragments of formononetin [34].

### 3.4. Screening Antioxidants of Alfalfa Flavonoids by DPPH-UHPLC

UHPLC was commonly used to conduct the qualitative and quantitative analysis of substances. In this study, all the conditions of UHPLC were the same as those of UHPLC-Q-TOF-MS/MS mentioned above. It was deduced that the content of antioxidants might be lower after they reacted with DPPH. Consequently, the effective antioxidants could be selected by the degree of peak area reduction [35]. The peak areas of fifteen compounds in 75% EEFs are shown in Table 2. As shown in Figure 3(b), it could be seen that all of the peak areas of flavonoids were decreased and presented different degree of decrease, which suggested fifteen compounds reacted with DPPH and their abilities to scavenge radical were different. The decrease rate of peak area of compounds 2 (hyperoside) and 4 (5,3′,4′-trihydroxyflavone) was decreased by 82.69% and 76.04%. It was indicated that their antioxidant abilities were stronger than those of the other thirteen flavonoids.

### 3.5. Effect of Alfalfa Flavonoids on Growth Performance and Mortality Rate of Broilers

The growth performance and mortality rate are presented in Table 3, and it could be observed that there was no significant difference among these four groups for body weight (BW) at 21 d of age; 0-28 d of age average daily gain (ADG), average daily feed intake (ADFI), and F : G; and mortality rate at 28 d, whereas the ADG and ADFI of three treated groups were higher than those of CN while the F : G of all treated groups were lower than that of CN. Particularly, the HDG showed the highest ADG and ADFI and the lowest F : G among all the groups. Moreover, BW and ADG at 56 d of age of LDG were higher than those of the other three groups (*P* < 0.05), and the 29-56 d of age of F : G was significantly lower than that of the CN (*P* < 0.01). However, there was no significant difference between the CN and the other two treated groups for 29-56 d of age of F : G and the mortality rate at 56 d of age of three treated groups sharply reduced.

### 3.6. Effect of Alfalfa Flavonoids on *In Vivo* Antioxidant in Broiler Serum

**Figure 4:**
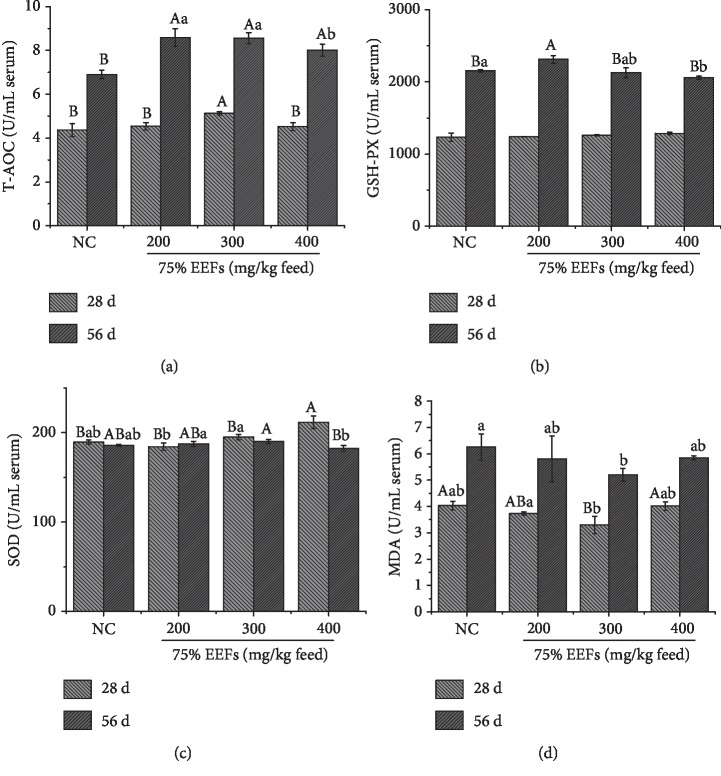
Effects of different dose of alfalfa flavonoids on GSH-PX, SOD, T-AOC, and MAD in broiler serum. Bars with different letters (a, b) were significantly different (0.01 < *P* < 0.05). Bars with different letters (A, B) were significantly different (*P* < 0.01). Data are means of 20 broilers for each treatment. NC = basal diet without 75% EEFs.

In the present study, four antioxidant indices in broilers treated with alfalfa flavonoids were analyzed. As shown in Figure 4, at the age of 28 d, the T-AOC of three treated groups were higher than that of NC, and the MDG significantly increased (*P* < 0.01). The GSH-PX of three treated groups was greater than that of NC. Compared with the NC, the SOD of HDG was obviously found to increase (*P* < 0.01). The MDA of three treated groups were lower than that of the NC, and the MDG significantly decreased (*P* < 0.01).

At the age of 56 d, the T-AOC of three treated groups significantly increased compared with the NC (*P* < 0.01). The GSH-PX of LDG and MDG were greater than that of NC. The SOD of MDG and LDG were found to increase (*P* < 0.01), and the HDG had no difference with the NC. The MDA of three treated groups were lower than that of the NC (*P* < 0.01).

## 4. Discussions

Notably, the total flavonoids, consisted of flavone, flavone C-glycoside, and flavonol, present powerful antioxidant activities. Recent papers have reported that flavonoids isolated from alfalfa could improve DPPH free-radical-scavenging activity, antioxidant activity, and growth performance [21, 36]. Antioxidants are a group of providing electrons or unpaired electrons. The O-phenolic group of flavonoids B ring poses as a hydrogen donor to receive free radicals and generated stable intramolecular hydrogen bonds with semiquinoid free radicals and thus blocking the free-radical chain reaction [37]. It has been reported that flavonoids can promote the proliferation and migration of adrenocortical stem cells and upregulate some growth hormones to improve animal growth and prevent the adverse effects of smoke exposure on bone by stimulating bone formation [38, 39]. Noticeably, our results indicated that 70% EEFs represented dramatically DPPH free-radical-scavenging activity and could improve the growth performance and *in vivo* antioxidant of broilers, whereas the complex interplay between alfalfa flavonoids and *in vivo* antioxidant involved broilers fed with the different dose of 75% EEFs for 28 and 56 d needed to further analysis.

Some studies reported that methanol had the highest extraction efficiency of flavonoids; ethanol was regard as ideal extraction solvent due to its higher safety and lower residue than other solvents. About 70% ethanol was commonly used to obtain the high content of flavonoids [40, 41]. Indeed, the content of 75% EEFs using ethyl acetate extraction was the highest among all the elution fractions. Hu et al. [42] reported that the TFC of ethyl acetate fractions was the highest level. Zhang et al. [43] found that the content of flavonoids varied with different EEFs, and the contents of 50% and 60% elution fractions were higher than those of any other fractions. In fact, the content and constitution of flavonoids were the two main factors in *in vitro* and *in vivo* antioxidant. Noteworthily, we noticed that the 75% EEFs was the high variety and content, which played a pivotal role in DPPH free-radical scavenging.

Briefly, we observed that fifteen flavonoids were isolated and identified. The 75% EEFs (200 *μ*g/mL) exhibited 95.51% DPPH free-radical-scavenging activity. Overwhelming evidence has demonstrated that the constitution of flavonoids was varied under different conditions, such as the content of extraction solvent, the cultivated varieties, harvest season, and extraction method [41]. Goławska et al. [44] researched the flavonoid concentration in aerial parts of three alfalfa varieties by HPLC and found that flavonoids of alfalfa are mostly glycosides of four flavone aglycones: apigenin, luteolin, tricin, and chrysoeriol. Interestingly, we noticed that five flavonoids were first reported, and apigenin, tricin, and chrysoeriol were also found in alfalfa. It was reported that the flavonoid structure, with C 2, 3 double bond of C ring and C 3-OH, C 4-OH of B ring, played an important role in scavenging free radical [45]. In addition, Von Gadow et al. [46] had reported that the formation of O-glycoside bond of C ring C 3-OH would reduce antioxidant capacities due to the lack of providing electron. Noticeably, the hyperoside and 5,3′,4′-trihydroxyflavone had a double bond and functional phenolic hydroxy group, whereas we speculated that might be the O-glycoside bond enhanced water solubility of hyperoside, and the reaction mechanism of antioxidants with different types of free radicals (lipid and hydroxyl free radicals) was different. However, the antioxidant activity of rutin (with two glycoside bonds) decreased due to the steric hindrance in our study.

Numerous studies have shown that bad environment leads to oxidative stress in organism, which increases the production of free radical that may cause damage to critical biomolecules including lipids, proteins, and DNA, and then result in reduced welfare, growth performance, and meat quality and increased tissue damage and bone loss [47]. Compelling evidence indicated that flavonoids could activate growth hormones to improve animal growth and prevent the adverse effects on bone by stimulating bone formation. Furthermore, considerable paper showed that flavonoids had positive effects on growth performance. Ma et al. [48] evaluated that the ADFI and BW of the treatment group (basal diet supplemented with flavones of sea buckthorn fruits) were higher than those of the control group. Ouyang et al. [20] demonstrated that alfalfa flavonoids could improve the ADG and BW of broilers. In the present study, alfalfa flavonoids increased the BW, ADG, and mortality rate, while decreased the F : G and mortality rate of broilers. At the age of 28 d, HDG had a better response than LDG and MDG. LDG might be effective in growth performance at the age of 56 d. We speculated that different broiler breeds had different responses to the dose of 75% EEFs.

Actually, flavonoids can not only scavenge free radical but also inhibit the formation of the superoxide ion and indirectly inhibit redox-sensitive transcription factors and prooxidant enzymes, and polyphenols can activate antioxidant enzymes by donating hydrogen from hydroxyl groups. Subsequently, the free-radical damage resulted from oxidation of lipids and other biological response was terminated [49]. As was known to all, SOD, T-AOC, and GSH-PX were the main antioxidant enzymes in organism and MDA was a dominant indicator of lipid peroxidation. Shi et al. [50] had reported the effects of alfalfa saponin extract (ASE) on growth performance and antioxidant activities. It was reported that ASE increased GSH-PX, CAT, and SOD activities in serum and tissues and a decreased MDA level. Dabbou et al. [51] reported alfalfa flavonoids could lower the MDA of rabbit plasma and liver, which was similar to our study. In our study, we observed 75% EEFs increased the T-AOC and SOD and decreased MDA of broiler serum. Noticeably, at the age of 56 d, the T-AOC and SOD of HDG had difference with the LDG and MDG of antioxidant indices, but there was no difference between HDG and LDG, MDG of GSH-PX, and MDA. We speculated that native chicken breeds had a different positive effect on different antioxidant indices during different growth stages.

## 5. Conclusions

The content of 75% EEFs was higher than that of 30% EEFs, and fifteen flavonoids were identified with UHPLC-Q-TOF-MS/MS in this study. In addition, hyperoside, liquiritigenin, limocitrin, 7,4′-dimethoxy-5-hydroxyflavanone, and isoliquiritigenin were isolated from alfalfa for the first time. The DPPH free-radical-scavenging capacity of 75% EEFs (200 *μ*g/mL) was equivalent to that of Vc. The 5,3′,4′-trihydroxyflavone and hyperoside displayed powerful DPPH free-radical-scavenging activities by calculating the decrease of peak area. At the age of 28 d and 56 d, three treated groups of GSH-PX, SOD, and T-AOC dramatically increased while MDA lowered in broiler serum. At the age of 29-56 d, the F : G of LDG sharply reduced. The survival rate of three treated groups significantly increased at the age of 56 d. Consequently, alfalfa flavonoids played an important role in antioxidant and growth performance.

## Figures and Tables

**Table 1:** Identification of flavonoid compounds in 75% EEFs from alfalfa.

No.	RT (min)	[M-H]^−^ (*m*/*z*)	Exact mass	Formula	Major product ions (*m*/*z*)	Proposed compounds	Compared with standard, literature, MassBank
1	22.10	609	610	C_27_H_30_O_16_	300	Rutin	The three of them
2	22.67	463	464	C_21_H_20_O_12_	300; 271; 255	Hyperoside	The three of them
3	25.37	447	448	C_21_H_20_O_11_	284; 255; 227	Astragalin	The three of them
4	26.57	269	270	C_15_H_10_O_5_	133; 135	5,3′,4′-Trihydroxyflavone	The MassBank
5	28.81	461	462	C_22_H_22_O_11_	283; 285; 255	Chrysoeriol-7-O-glucoside	The literature, MassBank
6	32.81	255	256	C_15_H_12_O_4_	135; 119; 91	Liquiritigenin	The literature
7	34.58	253	254	C_15_H_10_O_4_	135; 117; 91	7,4′-Dihydroxyflavone	The MassBank, standard
8	59.11	269	270	C_15_H_10_O_5_	117; 151	Apigenin	The literature, standard
9	62.29	299	300	C_16_H_12_O_6_	284; 256	Chrysoeriol	The literature, MassBank
10	63.02	329	330	C_17_H_14_O_7_	314; 299; 271	Tricin	The literature, MassBank
11	63.56	345	346	C_17_H_14_O_8_	330; 315	Limocitrin	The literature, MassBank
12	64.78	297	298	C_17_H_14_O_5_	282; 253	3′,7-Dimethoxy-3-hydroxyflavone	The literature, MassBank
13	66.82	267	268	C_16_H_12_O_4_	252; 223; 195	Formononetin	The literature, MassBank
14	67.04	255	256	C_15_H_12_O_4_	135; 119; 91	Isoliquiritigenin	The literature, MassBank
15	73.80	299	300	C_17_H_16_O_5_	284; 271; 256	7,4′-Dimethoxy-5-hydroxyflavanone	The MassBank

**Table 2:** The analysis of peak area with UHPLC and DPPH-UHPLC in 75% EEFs.

Peak no.	RT (min)	Areas of UHPLC	Areas of DPPD-UHPLC	Decrease rate of area (%)
1	22.10	1487.23	693.83	53.35
2	22.67	1982.41	343.15	82.69
3	25.37	849.87	424.19	50.09
4	26.57	2096.45	502.30	76.04
5	28.81	569.09	199.42	64.96
6	32.81	905.37	380.37	57.99
7	34.58	3847.21	2293.38	40.39
8	59.11	5149.34	2609.37	49.33
9	62.29	1239.22	442.20	64.32
10	63.02	2160.5	901.84	58.26
11	63.56	230.12	127.60	44.55
12	64.78	1131.28	417.63	63.08
13	66.82	635.56	509.12	19.89
14	67.04	987.4	458.00	53.62
15	73.80	12956.02	5753.88	55.59

**Table 3:** The growth performance and mortality rate of broilers at the age of 28 and 56 d.

Group	NC	LDG	MDG	HDG
Initial weight (g)	105.32 ± 0.53	105.52 ± 0.64	105.71 ± 0.60	106.06 ± 0.58
BW at 28 d of age (g)	538.10 ± 20.12	558.50 ± 26.32	560.72 ± 27.40	573.66 ± 31.33
BW at 56 d of age (g)	1276.01 ± 68.45^b^	1397.21 ± 75.09^a^	1311.20 ± 77.67^ab^	1320.00 ± 58.26^ab^
0-28 d of age ADG (g)	15.46 ± 0.37	16.18 ± 0.93	16.25 ± 0.98	16.70 ± 1.11
0-28 d of age ADFI (g)	35.51 ± 2.99	35.75 ± 3.80	36.37 ± 2.10	36.39 ± 4.72
0-28 d of age F : G	2.29 ± 0.10	2.21 ± 0.15	2.24 ± 0.09	2.17 ± 0.19
29-56 d of age ADG (g)	26.35 ± 2.57^b^	29.95 ± 2.44^a^	26.80 ± 2.61^ab^	26.55 ± 1.93^b^
29-56 d of age ADFI (g)	65.43 ± 6.20	61.78 ± 4.44	61.84 ± 4.63	61.58 ± 5.07
29-56 d of age F : G	2.48 ± 0.07^A^	2.07 ± 0.10^Bb^	2.32 ± 0.27^ABa^	2.31 ± 0.09^ABa^
Mortality rate at 28 d (%)	/	/	/	/
Mortality rate at 56 d (%)	6.66	/	/	3.33

^a,b^Means of 28 d and 56 d with no common superscript differ significantly (0.01 < *P* < 0.05). ^A,B^Means of 28 d and 56 d with no common superscript differ significantly (*P* < 0.01). Data are means of 20 broilers for each treatment.

## Data Availability

The data used to support the findings of this study are available from the corresponding author upon request.

## Supplementary Materials

Supplementary Materials Herein, we investigate whether antioxidant activity and growth performance of broiler serum can be improved by different content flavonoids from alfalfa. The results showed that 5,3′,4′-trihydroxyflavone and hyperoside exhibited stronger DPPH-scavenging capacities than other flavonoids and the level of SOD, T-AOC, GSH-PX was significantly increased and MDA was decreased by 75% EEFs. The F : G of broilers were lowered by 2.98-16.53%.
