# Supplementary figures and images for: Investigation of Chemical Composition, Antioxidant Activity, and the Effects of Alfalfa Flavonoids on Growth Performance
Source: Oxid Med Cell Longev. 2020 Feb 10;2020:8569237. doi: 10.1155/2020/8569237 (PMC7035581; doi:10.1155/2020/8569237)

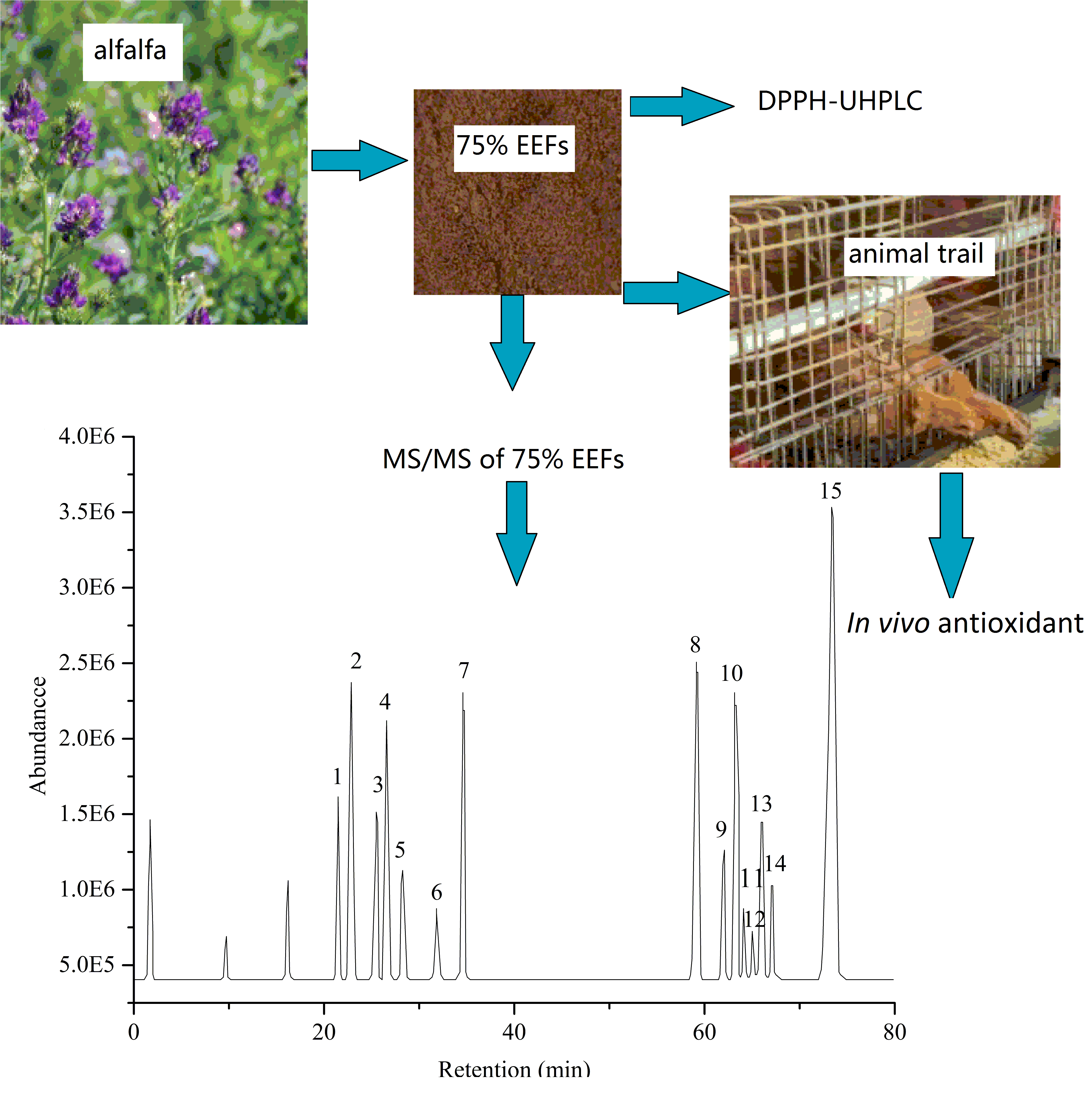

Supplement: Supplementary Materials — Herein, we investigate whether antioxidant activity and growth performance of broiler serum can be improved by different content flavonoids from alfalfa. The results showed that 5,3′,4′-trihydroxyflavone and hyperoside exhibited stronger DPPH-scavenging capacities than other flavonoids and the level of SOD, T-AOC, GSH-PX was significantly increased and MDA was decreased by 75% EEFs. The F : G of broilers were lowered by 2.98-16.53%. [file 8569237.f1.bmp]
